# Rule and Neural Network-Based Image Segmentation of Mice Vertebrae Images

**DOI:** 10.7759/cureus.27247

**Published:** 2022-07-25

**Authors:** Indeever Madireddy, Tongge Wu

**Affiliations:** 1 Computer Science, BASIS Independent Silicon Valley, San Jose, USA; 2 Department of Mechanical Engineering, University of California, Berkeley, USA

**Keywords:** machine learning, segmentation, image, mouse, bone

## Abstract

Background

Image segmentation is a fundamental technique that allows researchers to process images from various sources into individual components for certain applications, such as visual or numerical evaluations. Image segmentation is beneficial when studying medical images for healthcare purposes. However, existing semantic image segmentation models like the U-net are computationally intensive. This work aimed to develop less complicated models that could still accurately segment images.

Methodology

Rule-based and linear layer neural network models were developed in Mathematica and trained on mouse vertebrae micro-computed tomography scans. These models were tasked with segmenting the cortical shell from the whole bone image. A U-net model was also set up for comparison.

Results

It was found that the linear layer neural network had comparable accuracy to the U-net model in segmenting the mice vertebrae scans.

Conclusions

This work provides two separate models that allow for automated segmentation of mouse vertebral scans, which could be potentially valuable in applications such as pre-processing the murine vertebral scans for further evaluations of the effect of drug treatment on bone micro-architecture.

## Introduction

Image segmentation is the separation of an image into individual sections or regions [1]. These individual regions can then be further individually analyzed, allowing for more intricate observations to be made. Segmenting images is especially useful in the medical industry when analysis of complex or convoluted images is necessary [2]. An example of this is during clinical trials or during the regular course of treatment for drugs, therapies, or treatments that target bone health. Segmenting X-ray or computed tomography (CT) scans of bones allows scientists and researchers to better understand the effect a treatment has on bone density or structure. For example, image segmentation of the pelvic bone has aided greater information about prostate cancer metastasis during treatment [3]. Segmenting images also helps in screening and prediction. Image segmentation of the proximal femur allowed researchers to assess fracture risk [4]. Segmentation of CT scans was also vital for the prediction and detection of osteoporosis [5].

Vertebrae are especially challenging to segment because various vertebrae within a patient are of different sizes and compositions in addition to varying between patients as well [6]. Above all, vertebrae have a complex structure that is not easy to analyze [7]. This is because vertebrae are composed of two classifications of bone, namely, cortical and trabecular. These two bone styles are typically found in a 1:3 ratio, while around 80% of the bones in the rest of the human body are cortical [8]. Cortical bones form the outer shell of the vertebrae, providing the vertebrae with structure and surrounding the inner marrow space of the bone. Trabecular bones are often referred to as spongy bones that make up the internal structure of the vertebrae and of other bones of the body. Trabecular bones help redistribute the load experienced by the whole bone and overall help support the harder cortical bone [9]. It is challenging to segment vertebrae to separate trabecular and cortical sections because there is no clear boundary where one type of bone ends and another starts. There is no precise location where one can point to where the internal trabecular bone ends and the surrounding cortical bone starts. With the sharp rise in computational power and technology, machine learning models have been increasingly utilized as methods to segment images.

Prior works have examined how to semantically segment bone scans [10,11]; however, the methods employed tend to be complicated. These works take advantage of U-nets that can accurately semantically segment images with multiple convolutional layers. However, U-net neural networks have been previously characterized as requiring “considerable energy, memory bandwidth, and computer resources” [12]. Although U-nets continue to be useful for complex instance segmentation tasks, this work explores whether a simpler rule-based method or neural net could be as accurate as the existing U-net models for semantic segmentation. This would save computational resources and time while doing bone analysis. In short, this work aims to develop a new method in which vertebrae scans can be segmented. It is hypothesized that a linear-layer neural network will be able to provide comparable accuracy to the U-net networks currently in place. A rule-based model and a linear-layer neural network approach to segmenting mouse micro-CT images were explored and their functional accuracy was compared. The cortical shell of the vertebrae was segmented and isolated from the whole bone image, and model accuracy was predicted by comparing the model’s prediction of the cortical region to the actual region provided with the dataset. This work focuses on a supervised machine learning model because manually segmented images are available to train the neural network, thus avoiding the use of an unsupervised method like MIA clustering.

## Materials and methods

Dataset

**Figure 1:**
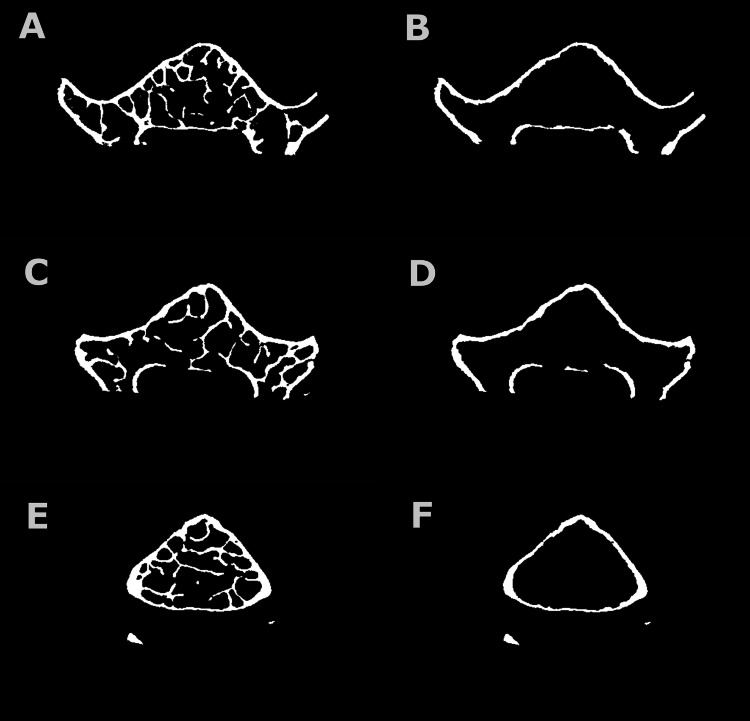
Total and cortical segmented vertebral micro-CT scan of a mouse vertebral body. Images A and B show partially open bone and the cortical shell, respectively, C and D show open bone scans, and E and F show closed scans. CT: computed tomography

Mouse micro-CT scans (Figure 1) were provided as a courtesy of Dr. Tony Keaveny of the University of California Berkeley, College of Engineering [13]. These scans were collected at the NASA Ames Research Center, and the collection was approved by the Institutional Animal Care and Use Committee under protocol #NAS‐13‐004‐Y3. The dataset analyzed in this work included 190 total images (Table 1) of mouse vertebrae scans and the corresponding predicted cortical segments. The images had dimensions 512 × 344 pixels and were binarized before being fed into the models. The Mathematica programming language (version 13.0.1) was chosen for this work due to its robust versatility in handling large image datasets in addition to its well-developed library of pre-built functions [14]. Data were imported into Mathematica locally with the in-built data loading functions. The images were again classified into the following three types: partially open, open, and closed. Scans that had a mix of closed regions and open regions within them were classified as partially open. Scans that had less than five closed regions were classified as open. Scans that had a fully connected cortical shell were classified as closed. This additional layer of classification allowed for more specific model tuning.

**Table 1:** Data classification. This table outlines how the 190 bone images were classified in this work.

Bone type	Number of images
Open	11
Partially open	88
Closed	91

Rule-based segmentation

First, a group of elementary functions (dilation, erosion, opening, closing) [15] built into the Mathematica software was explored to determine their ability to segment the cortical bone from the original bone image. These rule-based functions were used to create a mask that overlapped the cortical region in the bone scan. The mask would then be subtracted from the original image to isolate the trabecular region within the bone. Afterward, the trabecular regions would be again subtracted from the original image to isolate the cortical region. This cortical region would then be compared to the actual region provided in the dataset to calculate the error. Various combinations and permutations of the functions were manually studied to determine which set could generate an accurate mask for all the images in the dataset. However, it was found that no one set of functions could mask all the bones at once with high accuracy. Thus, the dataset was split manually into the following three types of bones: open, partially open, and closed. Three rule-based function sets were created, one for each type of bone. The percent error of a prediction was calculated by converting the images to matrices where elements corresponded to pixel values. Percent error was chosen as the accuracy metric as it allows for pixel-by-pixel analysis of the segmentation. A white pixel was assigned a value of 1, while a black pixel was given 0. The predicted cortical shell was numerically subtracted from the actual cortical region. The absolute value of the matrix was taken to account for both over and underestimates by the rule-based model. The number of white pixels was summed up and divided by the total number of white pixels in the actual cortical shell. Afterward, we also did an optimization study where we systematically swept six of the aforementioned functions and located their best range-r squared parameter for each function to reduce the error for each of the three bone types.

Neural network segmentation

**Figure 2:**
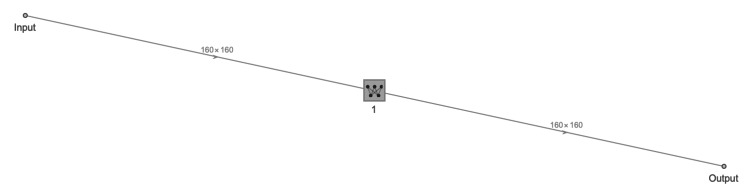
Linear layer neural network.

First, the dataset of images was split into testing and training sets. Various train-test splits were tested, with training incrementally increasing by 10%. The dataset included 190 images of various vertebral scans and their corresponding cortical shell. A linear neural network was then set up in Mathematica and trained on the pixel values behind the images and their corresponding cortical region. The network created an array of values and was tasked with determining whether a specific pixel would end up white or black in the segmentation. Each image was resized to 160 × 160 pixels and converted into an array of pixel values (either 0 or 1). This 160 × 160 array corresponding to each training image was fed to the linear layer neural network (Figure 2). The weight of the single layer was a matrix with a size of 25,600 × 25,600, and the bias of the layer was a vector of size 25,600.

**Figure 3:**
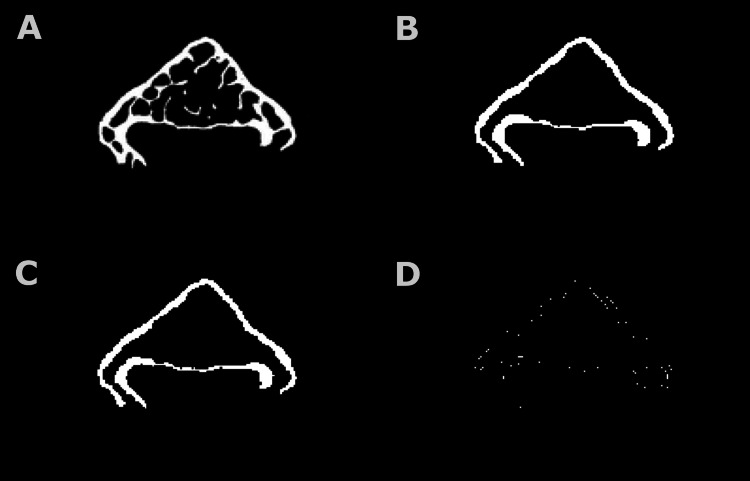
Neural-network-based segmentation of a closed bone scan. Image A depicts the original total bone. Image B shows the predicted cortical shell by the neural network. Image C depicts the actual cortical region. Image D depicts the error including the over or underestimates of the cortical region by the neural network.

After the model was trained with the training set, the neural network was applied to the testing set, and the neural network’s segmented results were compared to the correct results in the testing set. This is visually represented in Figure 3.

Similar to the rule-based model, the neural network model shows that having a higher training iteration does not necessarily provide the most accuracy in bone-type prediction. It is established that 80% training produces the highest accuracy while any higher percentage marginally reduces the prediction accuracy. The neural network was based on the Net Train function built into the Mathematica software. The neural network automatically chooses a loss function with the linear layer. It converts the bone image into a matrix of assigned dimensions and creates an output that is able to be converted back into a pixelated image. However, the images needed to be resized into smaller dimensions (160 × 160 pixels) due to computational memory constraints. Previous studies have also shown that numerical-based models like the neural network model provide a slight advantage in image-processing accuracy compared to classic image-based frameworks like the rule-based model. Buvari and Pettersson found that when assessing different deep learning models used to improve Alzheimer’s diagnosis, their numerical-based classifiers performed marginally better than the image-based classifiers [16]. However, a hybrid classifier they tested using both the image and numerical models provided for the highest accuracy of the three tools. Likewise, further testing on incorporating the rule-based model and neural network model into a hybrid machine learning tool may provide us with even better results. The rule-based model could be used first to initially process the image, with the neural network providing the final segmentation. This may be a way to solve the large computational requirements noted above for the linear neural network.

U-Net

**Figure 4:**
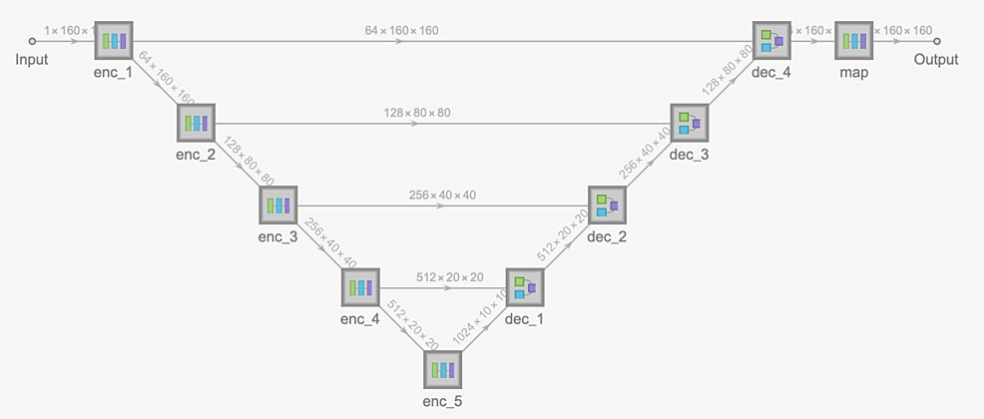
U-net.

A U-net was also trained on the same data as the neural network with the same test-train split. This net was based on the model developed by Hashmi and Goleshev [17]. Figure 4 visually depicts the U-net model, and Table 2 lays out its specifications. The same percent error metric used to calculate the accuracy of the neural network, and the rule-based model was used to determine the accuracy of the U-net.

**Table 2:** U-Net layer specifications.

Encoders/Decoders	Layer	Array Size
	Input	1 × 160 × 160
Encoder 1	Convolution layer	64 × 160 × 160
	Ramp	64 × 160 × 160
	Batch normalization layer	64 × 160 × 160
	Ramp	64 × 160 × 160
	Batch nortmalization layer	64 × 160 × 160
Encoder 2	Pooling layer	64 × 80 × 80
	Convolution layer	128 × 80 × 80
	Ramp	128 × 80 × 80
	Batch normalization layer	128 × 80 × 80
	Convolution layer	128 × 80 × 80
	Ramp	128 × 80 × 80
	Batch normalization layer	128 × 80 × 80
Encoder 3	Pooling layer	128 × 40 × 40
	Convolution layer	256 × 40 × 40
	Ramp	256 × 40 × 40
	Batch normalization layer	256 × 40 × 40
	Convolution layer	256 × 40 × 40
	Ramp	256 × 40 × 40
	Batch normalization layer	256 × 40 × 40
Encoder 4	Pooling layer	256 × 20 × 20
	Convolution layer	512 × 20 × 20
	Ramp	512 × 20 × 20
	Batch normalization layer	512 × 20 × 20
	Convolution layer	512 × 20 × 20
	Ramp	512 × 20 × 20
	Batch normalization layer	512 × 20 × 20
Encoder 5	Pooling layer	512 × 10 × 10
	Convolution layer	1024 × 10 × 10
	Ramp	1024 × 10 × 10
	Batch normalization layer	1024 × 10 × 10
	Convolution layer	1024 × 10 × 10
	Ramp	1024 × 10 × 10
	Batch normalization layer	1024 × 10 × 10
Decoder 1	Input 1	512 × 20 × 20
	Input 2	1024 × 10 × 10
	Output	512 × 20 × 20
Decoder 2	Input 1	256 × 40 × 40
	Input 2	512 × 20 × 20
	Output	256 × 40 × 40
Decoder 3	Input 1	128 × 80 × 80
	Input 2	256 × 40 × 40
	Output	128 × 80 × 80
Decoder 4	Input 1	64 × 160 × 160
	Input 2	128 × 80 × 80
	Output	64 × 160 × 160
Map	Convolution layer	1 × 160 × 160
	Logistic sigmoid	1 × 160 × 160
	Output	1 × 160 × 160

## Results

Rule-based model

We find that the rule-based model achieved an overall 72% accuracy in predicting the region of the cortical bone compared to the actual region provided in the dataset. This is based on a dataset of 190 bone images manually characterized as either partially open, open, or closed images. For bone scans classified as open, the model had a 69% accuracy. For partially open bones, accuracy dropped to 40%. Accuracy was significantly better for closed bones at 96% (Table 3). This accuracy was obtained after optimizing the parameters of the functions involved in the rule-based model to determine when overall accuracy was maximized. In essence, the optimal parameter is based on the number of iterations of the small and dark features on an image being removed or restructured. Some parameters such as geodesic opening were applied a varying number of times in each bone type. Table 4 outlines the parameters each type of bone was segmented with and the order in which the functions were applied.

**Table 3:** Accuracy of the rule-based and machine learning models.

Bone type	Rule-based model accuracy	Linear neural network accuracy	U-Net accuracy
Partially open	69%	97%	92%
Open	40%	96%	96%
Closed	96%	96%	97%

**Table 4:** Rule-based segmentation functions. The table depicts the functions in order applied to each bone type to segment it with the rule-based model. The number next to each function separated by a comma refers to the optimal parameter determined for that elementary function.

	Functions in order of application					
Bone character	1	2	3	4	5	6
Partially open	Blur, 10	Geodesic opening, 1	Geodesic Closing, 50	Binarize, 1	Edge detect, 1	Dilation, 4
Open	Erosion, 1	Geodesic opening, 3	Dilation, 1			
Closed	Dilation, 2	Geodesic closing, 20	Erosion, 5	Edge detect, 1	Dilation, 3	

Neural network model

The neural network model was significantly more accurate, with a 96% accuracy. Because the neural network was indiscriminately trained on all three bone types, the model was able to self-predict whether the input image was partially open, open, or closed and correctly segment the bone. A single network could be used on all three bone types. The neural network was incredibly consistent when tested on each type of bone individually, with a 97% accuracy on partially open bones, 96% accuracy on open bones, and 96% accuracy on closed bone scans. In each of these cases, the model was trained on all the bone types. To reiterate, the error was calculated by subtracting the prediction image from the actual image, counting the number of white pixels or the remaining, and then dividing by the total number of pixels. Accuracy then refers to the percentage of pixels that were predicted correctly. A 97% accuracy means that 97% of the white pixels in the prediction matched the pixels in the cortical image in the dataset. It can be seen that the linear layer neural network model had comparable accuracy to the U-net. Compared to the U-net, the neural network was more accurate at segmenting partially open bone scans, comparable when segmenting open scans, and only marginally less accurate when tasked with segmenting closed scans. The U-net’s lower accuracy for the partially open bone type could be attributed to the limited partially open bone scans available for training.

**Figure 5:**
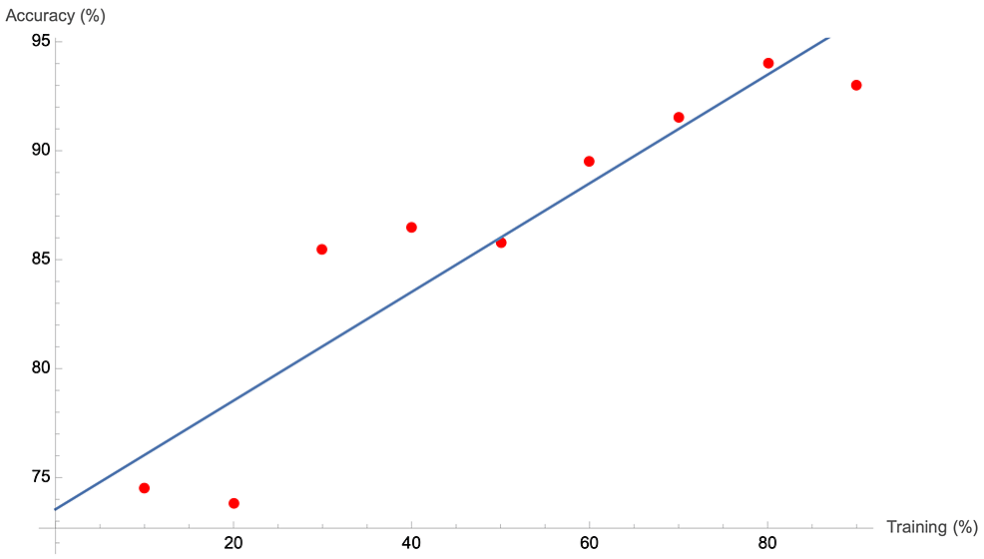
Accuracy versus percent of the dataset used to train the linear neural network. The line of best fit equation was 73.5 + 0.249× = y.

We also found a linear relationship between the amount of model training and the accuracy of image prediction for the neural network model; interestingly, however, any training over the 80% threshold reduces the accuracy, with an ideal 4:1 training to testing ratio in the dataset (Figure 5).

## Discussion

Rule-based model

For the rule-based model, there was no linear or direct relationship between the number of functional iterations (range-r squared parameter) and model prediction accuracy. This is clear because the same functions were iterated a different number of times for each bone type. Each iteration is denoted by the model removing small, dark features of the image. The function used and the parameter varied depending on the segmented bone type. Our findings suggest that the optimal number of iterations by the rule-based model required for the highest accuracy lies within the average of the total number of iterations among all three bone structures. It also seemed that the geodesic opening and geodesic closing functions, which rely on closed regions, were better suited to segmenting the similarly closed bone image scan. Hence, the closed structures, which lie in between the iteration extremes, produced the most accurate results, whereas the open and partially open bone types were either iterated too little or too much, respectively. This interpretation also assumes that the different bone types do not significantly impact how the models are run. A limitation of the rule-based model is its specificity to bone type which required multiple rule-based models to be developed to segment the three different bone types. Overall, the rule-based model was less flexible than the neural network, which could handle various bone types at once.

Neural network model

Through this current work, two models that may be potential alternatives for U-Nets have been explored. The rule-based model had an overall accuracy of 72%, while the linear neural network model had a 96% accuracy. The results show that the linear neural network was significantly more accurate. The neural network model was consistent within each bone type, while the rule-based model was significantly more inconsistent in its prediction accuracy of the three bone types. One interesting fact to note is that both the rule-based and neural network models had similar accuracies in predicting closed bone models (96%). This may be because of the predictable nature of closed bones. However, when predicting open or partially open bone scans, the accuracy of the rule-based model dropped drastically (40% and 69%). One cause of this may be the limited open bone scans (n = 11) available to develop the rule-based model. Closed bone scans also provide a smaller area for the models to predict, making it more likely that the model predicts correctly. Conclusively, the linear neural network is able to learn and better predict bone scans compared to the rule-based model and at a comparable level to the U-net architecture. Linear layer neural networks should be used as an alternative to the U-net models that currently exist for semantic segmentation. A limitation of this machine learning analysis was the limited data, especially partially open bone scans available to train the model. If more types of bone scans were available, the breadth of the neural network model could be increased.

## Conclusions

Our results provided evidence that a neural network was able to accurately segment the mice vertebral micro-CT scans. The neural network-based model was superior to the rule-based one and similar in accuracy to the U-net model. The applications of this work mainly reside in the medical industries, for example, aiding in the diagnosis of bone diseases or the treatment of them. However, other applications including analysis of predator-prey interactions, populations, and environmental conservation could also be conducted with improved image segmentation techniques. In short, this work provides a valuable alternative to the current U-net models and a straightforward computational method to segment micro-CT scans. Due to the versatility of machine learning, it will be straightforward to apply these models to human bones or to three-dimensional scans in the future.

## Appendices

Software code for this work is accessible through Github: www.github.com/IndeeverM/Neural-Network-Vertebrae-Segmentation.
